# Livestock Wastewater Treatment in Constructed Wetlands for Agriculture Reuse

**DOI:** 10.3390/ijerph17228592

**Published:** 2020-11-19

**Authors:** Sofia Dias, Ana P. Mucha, Rute Duarte Crespo, Pedro Rodrigues, C. Marisa R. Almeida

**Affiliations:** 1 CIIMAR—Interdisciplinary Centre of Marine and Environmental Research of the University of Porto, Terminal de Cruzeiros do Porto de Leixões, Avenida General Norton de Matos, 4450-208 Matosinhos, Portugal; sofiamdias5@gmail.com (S.D.); amucha@ciimar.up.pt (A.P.M.); 2 Chemistry and Biochemistry Department, Faculty of Sciences, University of Porto, Rua do Campo Alegre 687, 4150-171 Porto, Portugal; up201709923@fc.up.pt; 3 Biology Department, Faculty of Sciences, University of Porto, Rua do Campo Alegre 790, 4150-171 Porto, Portugal; up201603331@fc.up.pt

**Keywords:** phytoremediation, constructed wetland, wastewater, fertilizer

## Abstract

The aim of this study focused on the evaluation of constructed wetlands (CWs) microcosms, on a laboratory scale, for the removal of metals from a pig industry effluent while maintaining effluent organic matter and nutrients levels for its later used as a fertilizer. CWs with different macrophytes (*Phragmites australis* and *Typha latifolia*) and different substrates (light expanded clay aggregate and lava rock) were tested. Results showed high removals of metals during CWs treatment, with removal rates reaching >80% for Cd, Cr, Cu, Fe, Mn, and Zn after 2 days of treatment in CWs planted with *T. latifolia* and >60% in CWs planted with *P. australis*. Significant differences were only found between substrates for Fe and Mn in CWs with *P. australis*. Removal of organic matter (through chemical oxygen demand (COD)) was >77%, with no significant differences between substrates or plants. Removals of ammonium and phosphate ions ranged between 59–84% and 32–92%, respectively, in CWs with *P. australis* and 62–75% and 7–68% in CWs with *T. latifolia*, with no significant differences between substrates. Overall, CWs showed potential to be efficient in removing toxic contaminants, as metals, while maintaining moderated levels of nutrients, allowing the use of reclaimed water in agriculture, namely as fertilizer. If one aims for a short CW treatment, CW planted with *T. latifolia* and expanded clay as substrate could be the more suitable choice.

## 1. Introduction

Continued worldwide industrialization and exponential growth of the human population have led to an increase in wastewater generated. Wastewaters may contain different chemical compounds some of which can cause environmental and health risks [1]. Therefore, wastewater treatment is needed before its reuse.

Reusing treated wastewater represents an important part of sustainable water resource management and circular economy, reducing the use of freshwater [1]. Reclaimed water can be reused for different purposes depending on its quality and legislation in force. The higher use of reclaimed water is agricultural irrigation, because of the additional value of water due to the presence of fertilizers, nutrients, and macro elements [2,3,4,5,6,7]. An estimation indicates that water resources used for irrigation purposes is about 70% of that available in the world [6]. Therefore, the reuse of treated wastewater in the agricultural sector is of particular interest. However, as mentioned, wastewaters need to be adequately treated before its reuse to remove a wide variety of pollutants such as pesticides, pharmaceuticals, or metals [8]. In the case of livestock, metals and pharmaceuticals are the main contaminants of concern [9]. For instance, Zn and Cu are often used in high doses to enhance animals’ immune systems [10]. In fact, Zn salts have been used as an alternative to infeed antibiotics, which is believed to contribute to the emergence of methicillin-resistant *Staphylococcus aureus* [10,11]. The EU has banned the inclusion of pharmacological levels of ZnO after 2022. In Portugal, maximum levels of metals are legislated for fertilizers [12] and for wastewater discharges [13]. Recommend values for metals in the Portuguese legislation for irrigation water are identical or lower to those established in international legislation regarding water quality for irrigation [14,15]. Therefore, there is an emergence in remove/reduce these pollutants from livestock wastewaters.

A possible methodology to treat livestock wastewater is phytoremediation. Based on natural processes, phytoremediation is an efficient and extensively employed environmental cleanup biotechnology, that uses plants and associated microorganisms to remove, accumulate, metabolize, absorb and/or degrade organic and inorganic pollutants [9,16]. Phytoremediation has been shown to be an efficient process for removing metals from wastewater and this technology can have a key role when integrated in constructed wetlands (CWs) [9].

CWs have been reported to be efficient for the treatment of different types of wastewaters such as urban, agricultural, industrial, among others [17,18,19,20]. Livestock wastewaters tend to be more difficult to treat than urban wastewaters, due to their complex matrix and higher organic contents [21]. But several studies have shown the potential of CWs for the removal of different contaminants, including not only organic matter and nutrients but also biological contaminants, metals, or emergent pollutants (e.g., pharmaceuticals), from livestock wastewaters [8,10,21,22,23,24,25,26,27,28,29]. CWs are low-cost engineered systems designed to mimic natural wetland processes, using wetland vegetation, soils/substrate, and associated microorganisms to treat wastewater [8]. Pollutant removal is reached through a combination of physical, chemical, and biological processes in which all the elements of CWs, plants, microorganisms, and substrates, can have a significant influence [24]. Plants play an important role in CWs by uptaking pollutants, namely metals, by moving oxygen through their roots to the bottom of treatment wetlands, and by providing a medium beneath the water surface for the attachment of microorganisms that contributes to pollutant removal [30]. For instance, the use of microalgae or floating aquatic plants, such as duckweed, to phytoremediate livestock wastewater has been widely studied, because these plants can absorb organic matter and nutrients from water for their own growth needs [9]. Different macrophytes are also known to remove pollutants from water, such as *Juncus maritimus, Phragmites australis, Typha latifolia, Chrysopogon zizanioides, Colocasia esculenta*, among others [10,27,31,32,33]. *T. latifolia* and *P. australis* are two of the most commonly used plants in CWs, having properties of hyperaccumulation for different metals [30]. Substrates also have a significant role in CWs. Substrates support plants and microorganisms, promote biofilms formation, and improve the efficiency of CWs systems being involved in the adsorption, absorption, sedimentation, and filtration processes [30]. So, suitable substrates should be selected, as different layers and porous materials can have a positive impact on the removal of contaminants. Studies have shown that, for instance, light expanded clay aggregate and lava rock are appropriate for the development of plants and microorganisms in CWs [34,35,36].

In general, studies involving CWs aim the removal of all types of contaminants present in the wastewater to be treated. However, livestock wastewaters have a fertilizer potential, so removing toxic contaminants, such as metals, maintaining the nutritional value is a challenge. To our knowledge, there are no applications of CWs with this purpose. CWs offer a more sustainable and low-cost alternative to water treatment, so its potential should be explored.

The aim of this study focused on the evaluation of CWs microcosms simulating a vertical subsurface flow (VSSF) CW, on a laboratory scale, for the removal of metals from a pig industry effluent while maintaining effluent organic matter and nutrients levels to be later used as a fertilizer. To optimize the operation of the CWs, two types of porous substrate, lava rock and expanded clay, and two species of emerging macrophytes, *T. latifolia* and *P. australis*, which are among the most used plants in built wetlands [37], were tested. The final objective was to evaluate metals removal and nutrients permanence, so that nutrients final concentrations in the final effluents are suitable for fertilization, after the removal of the toxic contaminants.

## 2. Materials and Methods

Experiments were carried out in controlled conditions in six microcosms simulating CWs.

### 2.1. Sampling

Livestock wastewater was collected on a pig farm on the 14th and 29th of January and 13th of February of 2020. The wastewater was collected from a decantation lagoon used to store the liquid part of pig residues and was used as collected. An initial characterization was done, showing high levels of metals and nutrients (see Section 3).

*P. australis* plants were collected in River Lima Estuary (41.689822, −8.816289) in December of 2019 in Viana do Castelo, NW Portugal, and *T. latifolia* plants were collected in a riverside area near the coast in Leça da Palmeira, Matosinhos (41.205082, −8.715423) in February 2020. In the laboratory, plants were washed and replanted in each CW, ca. of 30 individual plants per microcosm for *P. australis* and ca. of 10 plants per microcosm for *T. latifolia,* as the latter have higher individual biomass.

### 2.2. Microcosms Assembly

Six CWs microcosms were assembled, as described previously [29], using plastic containers (40 cm × 30 cm × 30 cm) all with a first layer of gravel (3 cm) (Supplementary Material Figure S1). Three of the systems were filled with a second layer of lava rock (3 cm) (L systems: L1, L2, L3) and the other three with a second layer of lightweight expanded clay aggregate (A systems: A1, A2, A3). Finally, a third layer of sand (20 cm) was added to all systems, into which plants were transplanted. The first experiment was carried out with *P. australis*. At the end of this experiment, plants and sand were removed and the second experiment was initiated by transplanting *T. latifolia* into the same systems with a new sand layer.

All microcosms were wrapped in aluminum foil to prevent light penetration at a substrate level and possible photodegradation of the compounds. The wastewater was poured on top, to percolate through the different layers of the system, and drained out through a tap at the bottom when necessary, simulating a VSSF CW. Throughout the whole experience, wastewater was daily recirculated to prevent oxygen reduction in the system and avoid the development of anoxic microbiota. Microcosms were kept under greenhouse conditions, under normal light:dark regime and with a temperature variation between 9.3 °C and 32.3 °C.

### 2.3. Microcosms Operation

#### 2.3.1. Phragmites Australis

CW microcosms were firstly acclimatized for 6 weeks with a Hoagland nutrient solution in which solution was changed every two/three days, to reduce plant stress and allow the creation of rhizospheric microbiomes and biofilms in the systems. Afterwards, two stabilization weeks were performed (SW1 and SW2) in which livestock wastewater was added to the systems, to adapt plants to the load of organic and inorganic matter. In SW1, 2.5 L of wastewater plus 0.5 L of deionized water were added to each system. After one week, all water was removed from the CWs systems, and for SW2, 1 L of new wastewater plus 1.5 L of deionized water was added.

After stabilization, all water was removed from the CWs systems and the experiments started in which new livestock wastewater was added for a 15 days-cycle treatment (1 L of wastewater + 1.5 L of deionized water). Only 1 L of wastewater was added as SW1 showed that higher volumes of wastewater were not being properly treated with the wastewater still maintaining most of its smell and organic load, probably due to the small dimensions of the microcosms. This volume (2.5 L) was established to maintain the water level just above the substrate surface, allowing a complete saturation of the substrate (saturation rate of ca. 100%) [29]. During the first 7 days, samples were collected along the week for analysis of metals and nutrients (t1, t2, t3, t5, t6, and t7). At the end of this first cycle (t15), treated wastewater was removed and samples were collected for analysis of organic matter (through chemical oxygen demand (COD)), nutrients, and metal levels. New livestock wastewater was added and a second 15 days-cycle was initiated. Samples were collected after 7 days (t7.2) and 15 days (t15.2) and analyzed for the same parameters as the previously treated wastewater. All samples were collected by draining the liquid phase of each CW microcosm and adding deionized water to achieve the same volume as the initial added. So, the wastewater was changed every 15 days.

Water samples collected were stored at 4°C or −20°C depending on the parameter to be analyzed. Initial wastewater was also stored for analysis before being added to the systems. At the end of the experiments, all plants were removed from the systems, washed, and put to dry at room temperature until constant weight. Then, plants were separated into roots, rhizomes, leaves, and stems. Samples of sand in contact with the plant roots were also collected and dried at room temperature until constant weight.

#### 2.3.2. Typha Latifolia

CW microcosms were firstly acclimatized for one week with a Hoagland nutrient solution in which solution was changed every weekday, for plant establishment. Afterwards, CW microcosms were stabilized for 15 days (St) in which 950 mL of livestock wastewater plus 1.5 L of deionized water were added to each system.

After the stabilization, the wastewater was treated during two 15 day-cycles (1 L of wastewater + 1.5 L of deionized water). During the first 7 days of the first cycle, samples were collected along the week (t1, t2, t3, t6, and t7). At the end of the first cycle (t15), treated wastewater was removed and samples were collected for analysis of organic matter (through COD), metals, and nutrients. A new 15 days-cycle started, adding 1 L of new livestock wastewater plus 1.5 L of deionized water to each CW. During the first five days, water was not recirculated daily due to logistic constraints. Samples were collected after seven (t7.2) and fifteen days (t15.2) and analyzed for the same parameters as the previously treated wastewater. All samples were collected by draining the liquid phase of each CW microcosm and adding deionized water to achieve the same volume as the initial added. So, the wastewater was changed every 15 days.

Water samples were stored at 4 °C or −20 °C depending on the parameter to be analyzed. Initial wastewater was also stored for analysis before being added to the systems. At the end of the experiments, all plants were removed from the systems washed, and put to dry at room temperature until constant weight. Then, plants were separated into roots, rhizomes, and leaves. Samples of sand in contact with the plant roots were also collected and dried at room temperature until constant weight.

### 2.4. Samples Analysis

All reagents used were pro analysis or equivalent. All material was washed with deionized water (conductivity < 0.1 μS cm^−1^), immersed in nitric or chloric acid solution (20% *v/v*) for 24 h, washed again with deionized water, and dried in a clean oven.

For metals, initial livestock wastewaters, sand samples, and plant tissues (dry samples) were digested at high pressure with concentrated nitric acid (HNO_3_) and hydrogen peroxide (H_2_O_2_) solution. Treated wastewater samples were only acidified (with 1% HNO_3_) before direct analysis. Digestions were carried out in a high-pressure microwave system (Ethos, Milestone, Sorisole, Italy) in closed Teflon vessels. Metals (Cd, Cr, Cu, Fe, Mn, Ni, Pb, and Zn) were measured as described in previous studies [27], by atomic absorption spectrophotometry with flame atomization (AAnalyst 200 PerkinElmer, PerkinElmer Inc., Waltham, MA, USA) or electrothermal atomization (4100ZL, PerkinElmer, PerkinElmer Inc., Waltham, MA, USA) depending on metal levels. A calibration curve obtained with aqueous standard solutions of different metal concentrations (0–3 mg/L) was used for metal quantification. These standard solutions were prepared from 1000 mg/L stock standard solutions of each metal. Each individual sample was analyzed in triplicate with relative standard deviation always below 5%. For quality control, doped samples were prepared and analyzed with recovery values always between 80–120%. The detection limits (LOD) were 0.05 mg/L for Cu, Zn and Mn, 0.3 mg/L for Fe, 6.6 µg/L for Pb, 0.6 µg/L for Cd, 2.5 µg/L for Ni and 1 µg/L for Cr.

COD was measured using the Kits HI93754B-25 (Hanna Instruments, Padua, Italy), MR from 0 to 1500 mg/L and following provided protocol.

For nutrients, all samples were filtered (nitrate cellulose filters, 0.45 μm porosity). Dissolved ammonium, nitrate, nitrite, and phosphate ions concentrations were determined following the methods described in [8]. Nitrate was quantified by an adaptation of the spongy cadmium reduction technique [38], evaluating the reduction percentage to nitrites. All the analyses were performed in triplicate. The detection limits were 0.05 mg/L for ammonium and phosphate ions, 0.1 mg/L for nitrate ions and 0.01 mg/L for nitrite ions. For quality control, doped samples were prepared and analyzed with recovery values always between 80–120%.

### 2.5. Statistical Analysis

All parameters in wastewaters were measured in triplicate (three independent microcosms) and means and standard deviations were calculated for each treatment (*n* = 3). To evaluate statistically significant differences (*p* < 0.05), t-student tests and a parametric one-way analysis of variance (ANOVA) were applied. The presence of significant differences was detected by a multiple Tukey comparison test.

## 3. Results

To assess the functionality of CWs over time, analyzes of livestock effluent quality parameters (metals, nutrients, and organic matter content) were performed before and after CW treatment.

During the experiment, some changes in the visual aspect of the plants were observed. Initially, they were well-nourished and alive; however, at the end of the experiment, they were visually weak and malnourished indicating a negative impact of the wastewater.

### 3.1. Metals

#### 3.1.1. Water

##### Phragmites Australis

In the experiments with *P. australis*, concentrations of each metal in the initial livestock wastewater were in the order Fe > Zn > Mn > Cu > Ni > Pb > Cr > Cd (Supplementary Material—Table S1). Initial concentrations of Cr, Ni, and Pb were the only ones already lower than the legislated recommended value for irrigation water and legislated limit for wastewater discharge (Supplementary Material—Table S6) [13,14]. Initial concentrations of Cd, Cu, Mn, and Zn were lower than the legislated maximum allowable value for irrigation water [13], but Fe concentrations were higher. Cd initial concentration was also lower than the legislated limit for wastewater discharge [13], but Cu, Mn, and Fe were higher (no legislated values for Zn).

**Figure 1:**
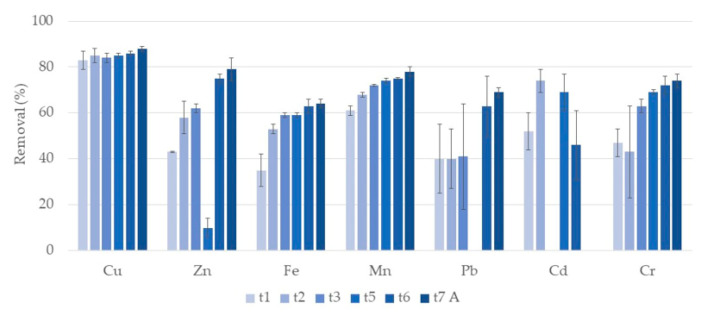
Removal percentage and respective standard deviation (*n* = 3) for each metal, in constructed wetlands (CWs) with expanded clay (A) and *P. australis* during the first week of treatment. Samples taken after one day (t1), two days (t2), three days (t3), five days (t5), six days (t6) and seven days (t7) of treatment. At t5 there was no removal for Pb. For Cd no removal was observed at t3.

Figure 1 shows the removal rates of the different analyzed metals during the first week of treatment in systems with clay as the porous substrate (A systems). For Cu and Pb no significant differences among removals were observed. For Pb, removals tended to increase but with a high variability among microcosm replicates. Removal rates ranged between 83–88% during the week for Cu and between 40–69% for Pb. Zn, Fe, Mn, and Cr had significant increasing removals during the week, achieving removals of 79%, 64%, 78%, and 74% after 7 days of treatment. Cd showed a different behavior, with removals increasing and decreasing during the week, ranging between 46–74%. For Ni, in general, no removal during the week was observed. Concentrations of Fe and Zn after two days of treatment were already lower than legislated recommended value for irrigation water [13,14]. However, after 7 days, concentrations of Cd, Cu, and Mn were still higher than the recommended value. Regarding wastewater discharge legislation [13], after one day of treatment, concentrations of Cu and Mn were already lower, but concentrations of Fe were still higher than legislated value after seven days of treatment.

**Figure 2:**
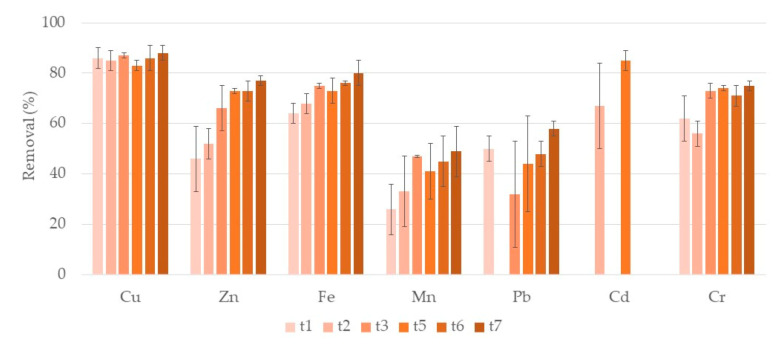
Removal percentage and respective standard deviation (*n* = 3) for each metal, in CWs with lava rock (L) and *P. australis* during the first week of treatment. Samples taken after one day (t1), two days (t2), three days (t3), five days (t5), six days (t6), and seven days (t7) of treatment. For Pb, there was no removal at t2 and for Cd, there were no removals at t1, t3, t6, and t7.

Figure 2 shows the removal rates of each metal during the first week of treatment in systems with lava rock as the porous substrate (L systems). For Cu, Mn, Pb, and Cd no significant differences along the week were observed. During the week, removal rates ranged between 83–88% for Cu, 26–49% for Mn, 32–58% for Pb, and 67–85% for Cd, although for Cd it varied greatly during the week. Zn, Fe, and Cr showed significant increasing removals over the week, achieving values of 77%, 80%, and 75%, respectively, at t7. Once again, no removal of Ni was observed. Concentrations of Fe, Cd, and Zn after one, two, and three days of treatment, respectively, were already lower than legislated recommended value for irrigation water [13]. Concentrations of Cu and Mn were still higher than the recommended value after seven days of treatment. Regarding wastewater discharge legislation, concentrations of Cu, Mn, and Fe, after one, three, and seven days of treatment, respectively, were already lower.

**Figure 3:**
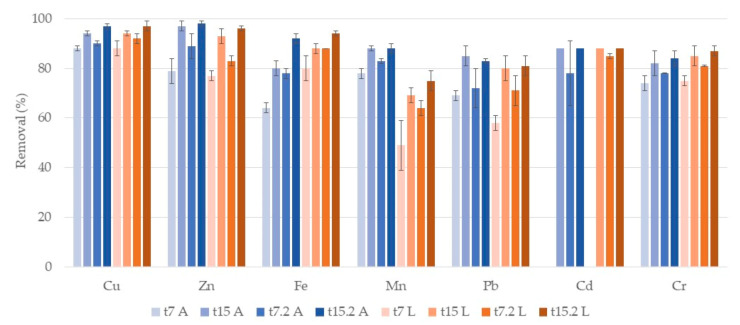
Removal percentage and respective standard deviation (*n* = 3) for each metal, in CWs with *P. australis* and expanded clay (t7 A, t15 A, t7.2 A, t15.2 A) and lava rock (t7 L, t15 L, t7.2 L, t15.2 L) over the two cycles of treatment. First cycle: samples taken after one week (t7) and at the end of the two-week cycle (t15). Second cycle: samples taken after one week (t7.2) and at the end of the two-week cycle (t15.2). For Cd, there was no removal at t15.2 A.

Figure 3 shows a comparison in terms of removal of both substrates (A and L) in the two cycles of treatment. For Cu, Zn, and Pb no significant differences between substrates were observed. It is possible to notice, for these metals, an increase in removal in the second week of treatment (t15 A, t15.2 A, t15 L, t15.2 L) in both systems in comparison to the first week (t7). So, a 15-day cycle could be more appropriate to remove a higher metal level.

For Fe at t7 and t7.2 a significant difference between substrates was observed, with L systems removing more efficiently Fe after one week of treatment. However, at t15 and t15.2 no significant differences between substrates occurred. The second week of treatment had also higher removals than the first week, in both cycles for both substrates. Moreover, for both systems, a difference between cycles of treatment was observed. The second cycle showed higher removals than the first one, for both substrates. For Mn, significant differences between substrates were also observed for both cycles, but A systems were more efficient in removing Mn than L systems. In the second week of treatment higher removal values were achieved. For this metal, no differences between cycles were observed. For Cr, no significant differences between substrates, or between cycles, were observed. Besides, no significant differences between one and two weeks of treatment were also observed, which suggests that one-week treatment is efficient in removing this metal. At t7 no removals of Cd in either system occurred and at t15 and t15.2 Cd concentrations were below the limit of detection (LOD = 0.6 mg/L), so removals were >88%, without differences between substrates.

As mentioned, for Cd, Cu, Zn, Fe, Mn, and Pb a two-week treatment is needed to remove a higher amount of metals. Nevertheless, concentrations after one week (t7 A, t7.2 A, t7 L, t7.2 L) were lower than the maximum allowed levels for irrigation water for all analyzed metals [13], with the exception of Mn, Cu, and Cd in the first cycle that required two-week treatment. In general, both substrates were equally efficient, although there were some differences for Fe and Mn. For Ni, no removals were observed, initial concentration was 126 ± 23 µg/L and at the end of the first cycle, they were of 159 ± 7 µg/L and 143 ± 10 µg/L for A and L systems, respectively. Initial concentration in the second cycle was 124 ± 12 µg/L and at the end, they were 132 ± 14 µg/L and 113 ± 9 µg/L. These values were lower than legislated ones [13,14].

#### Typha Latifolia

In the experiment with *T. latifolia*, the measured concentrations of each metal, in the initial waters were in the order Fe > Zn > Mn > Cu > Ni > Cr > Pb > Cd (Supplementary Material Table S1). Initial concentrations of Cd, Cr, Ni, and Pb were the only ones with already lower than the legislated recommended value for irrigation water and legislated limit for wastewater discharge [13,14]. Initial concentrations of Cu, Mn, and Zn were lower than the legislated maximum allowable value for irrigation water [13], but Fe concentrations were in some cases higher. Cu and Mn initial concentrations were lower than the legislated limit for wastewater discharge [13], but Fe concentrations were higher (no legislated values for Zn).

**Figure 4:**
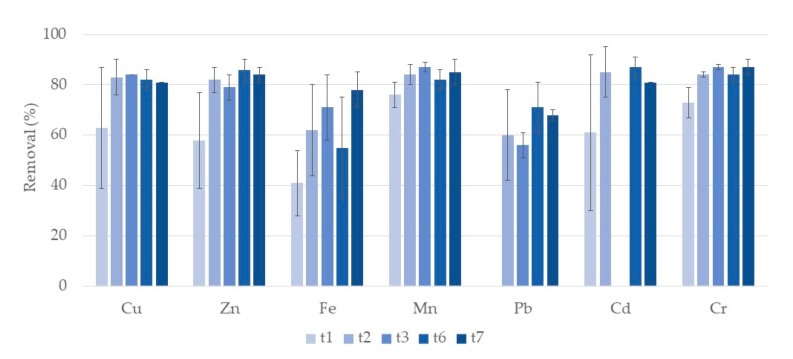
Removal percentage and respective standard deviation (*n* = 3) for each metal, in CWs with expanded clay (A) and *T. latifolia* during the first week of treatment. Samples taken after one day (t1), two days (t2), three days (t3), five days (t5), six days (t6), and seven days (t7) of treatment. There was no removal of Cd at t3.

Figure 4 shows the removal rates of the different analyzed metals during the first week of treatment in A systems. For Cu, Zn, Fe, Mn, Pb and Cd no significant differences among removals along the week were observed. During the week, removal rates ranged between 63–84% for Cu, 58–86% for Zn, 41–78% for Fe, 76–87% for Mn, 56–71% for Pb, and 61–87% for Cd, although once again there were variations for Cd. For Cr removals increase from the first day (t1) to the following days, achieving a removal of 87% at t7. For Ni, in general, no removal was observed. Concentrations of Fe and Zn after one day of treatment were already lower than legislated recommended value for irrigation water [13,14]. Concentrations of Cu and Mn were higher than the recommended value for irrigation water after seven-day treatment. In concern of wastewater discharge legislation, after three days of treatment concentrations of Fe were already lower.

**Figure 5:**
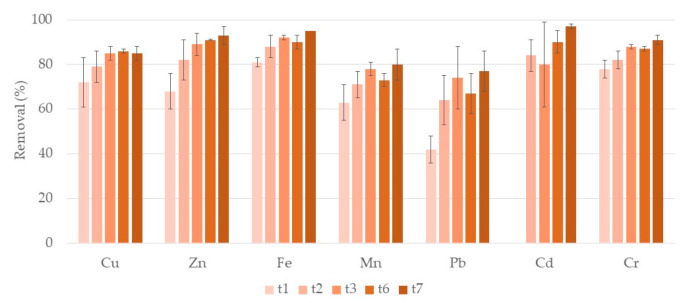
Removal percentage and respective standard deviation (*n* = 3) for each metal, in CWs with lava rock (L) and *T. latifolia* during the first week of treatment. Samples taken after one day (t1), two days (t2), three days (t3), five days (t5), six days (t6), and seven days (t7) of treatment. For Cd, there was no removal at t1.

Figure 5 shows the removal rates of each metal during the first week of treatment in L systems. For Cu and Cd, no significant differences among removals along the week were observed. Removal rates ranged between 72–86% during the week for Cu and between 80–91% for Cd. Zn, Fe, Mn, Pb, and Cr showed an increase between the first day (t1) and the following days, achieving removal rates of 93%, 95%, 80%, 77%, and 91%, respectively, at t7. Concentration of Fe at t7 was below the LOD of 0.3 mg/L, so removal was >95%. Once again, no removal of Ni was observed. Concentrations of Fe and Zn after one day of treatment were already lower than legislated recommended value for irrigation water [13,14]. Concentrations of Cu and Mn concentrations were still higher than the recommended value after seven-day treatment. In concern of wastewater discharge legislation, after one day of treatment, concentrations of Fe were already lower than the legislated limit.

**Figure 6:**
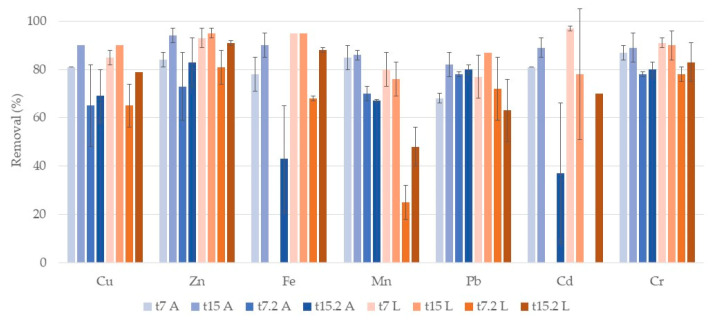
Removal percentage and respective standard deviation (*n* = 3) for each metal, in CWs with *T. latifolia* and expanded clay (t7 A, t15 A, t7.2 A, t15.2 A) and lava rock (t7 L, t15 L, t7.2 L, t15.2 L) over the two cycles of treatment. First cycle: samples taken after one week (t7) and at the end of the two-week cycle (t15). Second cycle: samples taken after one week (t7.2) and at the end of the two-week cycle (t15.2). At t7.2 A there was no removal of Fe and Cd, and at t7.2 L there was no removal of Cd.

Figure 6 shows a comparison in terms of metal removal of both substrates (A and L) in the two cycles of treatment. For Cu in A systems, no significant differences between cycles were observed, but in L systems removal rates decreased in the second cycle. In general, no significant differences between the first and second week of treatment in both cycles, or between substrates, were observed. Concentrations of Cu at t15 (A and L) and t15.2 L were below the LOD of 0.05 mg/L, so removals were > 90% for t15 and >79% for t15.2. For Zn and Pb, also no significant differences between the first and second week of treatment, or between cycles, were observed. No significant differences between substrates were either observed for these metals. At t15 L, concentrations of Pb were below the LOD of 6.6 mg/L so removal was >87%.

For Fe differences between cycles occurred, with lower removals in the second cycle. Comparing the removals of the first and second weeks, there were differences between t7 A–t15 A and t7.2 L–t15.2 L. At t15 no differences were observed between substrates. No removal of Fe occurred at t7.2 A and concentrations at t7 L and t15 L were below the LOD of 0.3 mg/L, so removals were >95%. For Mn significant differences between cycles were observed, with the second cycle showing lower removals. In general, differences between the first and second weeks of treatment were not observed. In the first cycle, there were no significant differences between substrates, but in the second cycle, Mn removal rates were lower in L systems. For Cd, significant differences between cycles occurred, with the second cycle showing lower removal rates. At t7.2 no removals of Cd were observed in either system. In the first cycle, no significant differences were observed between the first and second weeks. Additionally, no significant differences were found between substrates. For Cr, no significant differences between weeks of treatment or differences between substrates were observed. Between cycles, differences were found at t7 and t7.2 in both substrates but at t15 and t15.2 no significant differences were found.

For this plant also, in general, both substrates were equally efficient although some differences for Fe and Mn occurred. Contrarily to what was observed for *P. australis*, in general, no significant increase on metal removal was observed in the second week of each cycle, with values being in general below the recommended value for irrigation water. But for Cu values were lower than the recommended value for irrigation water only after a two weeks treatment. For Mn, even after a two-week treatment values were still higher than legislated ones. For Ni, no significant removals were observed: initial concentration was 83 ± 22 µg/L and at the end of the first cycle they were of 80 ± 5 µg/L and 47 ± 6 µg/L for A and L systems, respectively. Initial concentration in the second cycle was 59 ± 5 µg/L and at the end, Ni concentrations were 84 ± 5 µg/L and 55 ± 9 µg/L. These values were lower than legislated ones [13,14].

#### 3.1.2. Plant Tissues

For *P. australis*, in general, the initial metal concentrations in leaves, stems, roots, and rhizomes were higher than the concentrations measured at the end of the experiment except for Fe, Mn, and Zn in some plant tissues (Supplementary Material—Table S2). Mn was the only metal accumulated in all plant tissues, with accumulation values between 47% and 64%, depending on the plant tissue. Zn showed a high accumulation in stems, 76% and 75% for A and L system, respectively, and leaves, 60% and 68% for A and L system, respectively (Supplementary Material—Tables S3 and S4). No Zn accumulation in below-ground tissues was observed. Fe was only accumulated in plant leaves, 78% and 64% for A and L system, respectively.

In *T. latifolia*, the concentrations of metals at the end of the experiment were equal or lower than the initial values, indicating that metals were not accumulated in any of tissue plants (Supplementary Material—Tables S2–S4).

In general, no significant differences were observed between substrates.

Both plant species showed signs of toxicity due to the progressive appearance of dry leaves, with the aerial part of *P. australis* ending up dying at the end of the experiment.

#### 3.1.3. Sand Substrate

In the initial sand, concentrations of Cu, Mn, Ni, and Zn were below the detection limit (LOD = 4 µg/g for Cu, Zn, and Mn and 0.2 µg/g for Ni), whereas Cd, Cr, and Pb were detected showing concentrations of 0.7 ± 0.1 µg/g, 0.9 ± 0.3 µg/g and 13 ± 8 µg/g, respectively. (Supplementary Material —Table S2). Metals levels in sand collected at the experiment with *P. australis* only showed accumulation of Mn in L systems, of 40% (for A system concentration was identical to initial ones). Concentrations of Cr, Cu, Fe, Ni, and Zn showed no significant differences comparing to the initial sand, whereas concentrations of Cd were below the detection limit (LOD = 0.05 µg/g) and of Pb were 1.0 ± 0.3 µg/g and 1.7 ± 0.5 µg/g for A and L systems, respectively.

Metals levels in sand collected at the experiment with *T. latifolia* only showed accumulation of Mn, ca. of 65% for A and 44% for L systems. Concentrations of Cu, Fe, Ni, and Zn showed no significant differences to the initial sand. Concentrations of Pb were below the detection limit (LOD = 0.5 µg/g). Concentrations of Cd and Cr showed no significant differences in A systems and were below the detection limit (LOD = 0.5 µg/g for Cd and 0.08 µg/g for Cr) in L systems.

### 3.2. Nutrients

The rates of nutrient removal from swine wastewater were calculated and evaluated.

#### 3.2.1. Phragmites Australis

**Figure 7:**
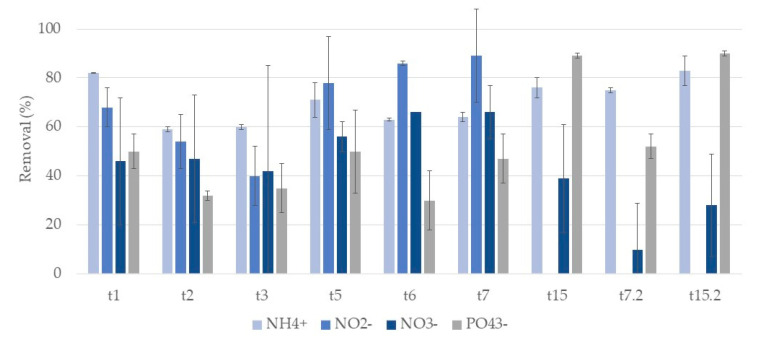
Removal percentage and respective standard deviation (*n* = 3) for each nutrient ion, in CWs with expanded clay (A) and *P. australis* during the two cycles of treatment. First cycle: samples taken after one day (t1), two days (t2), three days (t3), five days (t5), six days (t6), seven days (t7), and at the end of the two-week cycle (t15). Second cycle: samples taken after one week (t7.2) and at the end of the two-week cycle (t15.2) of treatment. At t6, only one replicate was considered for nitrate ion.

**Figure 8:**
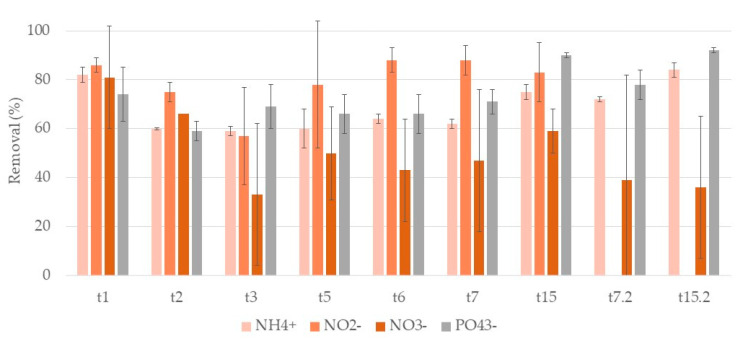
Removal percentage and respective standard deviation (*n* = 3) for each nutrient, in CWs with lava rock (L) and *P. australis* during the two cycles of treatment. First cycle: samples taken after one day (t1), two days (t2), three days (t3), five days (t5), six days (t6), seven days (t7), and at the end of the two-week cycle (t15). Second cycle: samples taken after one week (t7.2) and at the end of the two-week cycle (t15.2) of treatment. At t2, only one replicate was considered for nitrate ion.

Figure 7 and Figure 8 show the removal rates of ammonium, nitrite, and phosphate ions in systems A and L, respectively.

For ammonium ion, removals show no clear tendency to increase or decrease during the week. At t7 and t15 no significant differences were observed between substrates, with removal percentages of 64% and 76% respectively for A systems, and 62% and 75% for L systems. The second cycle showed higher removal rates, 75% and 83% at t7.2 and t15.2 for A systems and 72% and 84% for L systems.

Nitrite ion removals oscillated during the first week in both systems, with no significant differences between substrates. A systems achieved a removal of 89% at t7 and showed no removals at t15. L systems showed removals of 88% and 83% at t7 and t15, respectively. In the second cycle, no removals were observed in both systems, with nitrite ion concentration increasing.

Regarding nitrate ion, removals were very variable during the first week for both substrates. In general, L systems showed higher removals rates. For A systems, at t7 and t15 removals of 66% and 39% were observed and for L systems removals were 47% and 59%. The second cycle showed lower removal rates for both systems. For A systems removals were 10% and 28% at t7.2 and t15.2 for L and 39% and 36%. Concentrations of nitrate ion were very variable, even in replicate microcosm CWs.

For phosphate ion, removals vary throughout the first week, with no clear tendency to increase or decrease in both systems. During the first week (from t1 to t7), in general, L systems showed higher removal rates than A systems, achieving 71% at t7, while A systems presented a removal of 47%. At the end of the second week (t15), no differences between the substrates were observed, being 89% for A systems and 90% for L. The second cycle was similar to the first, showing removals of 52% and 90% at t7.2 and t15.2 for A systems and 78% and 92% for L systems.

So, in general, no influence of the substrates in nutrient ions removals was observed. Moreover, nutrient removal occurred mainly on the first day, and no significant increments were observed during the following days of treatment. In addition, no significant differences were observed between cycles.

#### 3.2.2. Typha Latifolia

**Figure 9:**
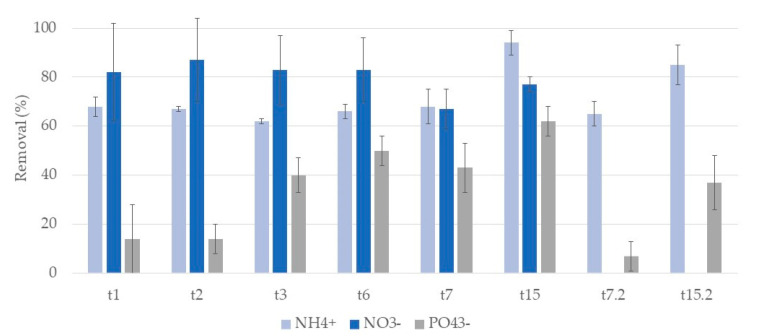
Removal percentage and respective standard deviation (n = 3) for each nutrient ion, in CWs with expanded clay (A) and *T. latifolia* during the two cycles of treatment. First cycle: samples taken after one day (t1), two days (t2), three days (t3), five days (t5), six days (t6), seven days (t7), and at the end of the two-week cycle (t15). Second cycle: samples taken after one week (t7.2) and at the end of the two-week cycle (t15.2) of treatment.

**Figure 10:**
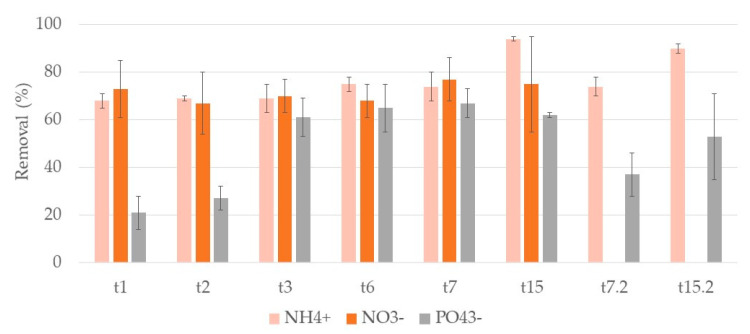
Removal percentage and respective standard deviation (*n* = 3) for each nutrient ion, in CWs with lava rock (L) and *T. latifolia* during the two cycles of treatment. First cycle: samples taken after one day (t1), two days (t2), three days (t3), five days (t5), six days (t6), seven days (t7) and at the end of the two-week cycle (t15). Second cycle: samples taken after one week (t7.2) and at the end of the two-week cycle (t15.2) of treatment.

Figure 9 and Figure 10 show the removal rates of ammonium, nitrate, and phosphate ions during both cycles of treatment for systems A and L, respectively.

Regarding ammonium ion results, during the first week, removals rates ranged between 62–68% for A systems and 68–75% for L systems. At t15 removals increase with values of 94% for both systems. The second cycle was similar to the first, removals of 65% for A systems and 74% for L systems at t7.2 and of 85% and 90% at t15.2, respectively. No significant differences were found between substrates.

For nitrite ion, no removals were observed in both cycles. The initial concentration was 5.3 ± 0.4 mg/L (Supplementary Material—Table S5), and at the end of the first cycle, concentrations were 7 ± 5 mg/L and 11 ± 4 mg/L for A and L systems, respectively. In the second cycle, initial nitrate ion concentration was 1.3 ± 0.2 mg/L, and at the end of the cycle, they were 10 ± 3 mg/L and 10 ± 6 mg/L for A and L systems, respectively.

Nitrate ion concentrations were monitored showing positive removal results in the first cycle, while in the second cycle the results were inconclusive as they did not show significant removals over time. In the second cycle, initial nitrate ion concentration was 8 ± 1 mg/L, and at the end of the cycle were 24 ± 5 mg/L and 19 ± 5 mg/L for A and L systems, respectively. The initial concentration of nitrate ion in the second cycle was ca. ten times lower than the initial concentration in the first cycle, which may explain the differences between removals in the two cycles. In the first cycle, nitrate ion removals ranged between 67–87% for A systems and 67–88% for L systems. No significant differences were found between substrates.

For phosphate ion, in A systems, the removal increased, during the first cycle, being 44% and 62% at t7 and t15, respectively. For L systems, removals slightly increased, reaching 68% and 62% at t7 and t15, respectively. No significant differences between substrates were observed. In the second cycle, removals increased from the first week to the second in A systems, but with lower removal rates than in the first cycle. L systems showed no significant differences between t7.2 and t15.2. A systems showed 7% and 37% removal rates at t7.2 and t15.2, and L systems showed 37% and 54% removals at t7.2 and t15.2.

So, for this plant also no significant differences were observed when comparing the two substrates. For *T. latifolia,* nutrient ions removal in general increased over time.

### 3.3. Organic Matter

**Figure 11:**
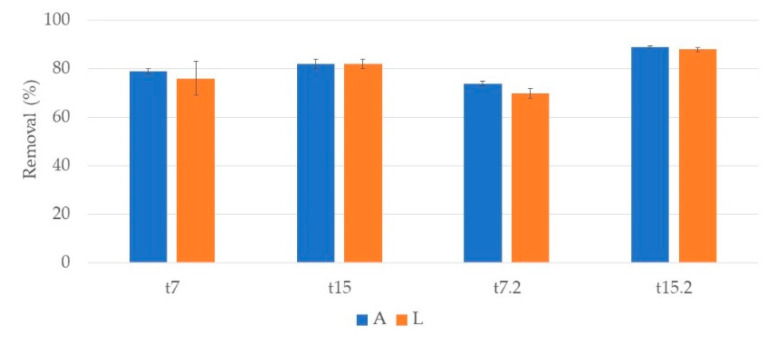
Removal percentage and respective standard deviation (n = 3) of COD, in CWs with *P. australis* and expanded clay (A) or lava stone (L) over the two cycles of treatment. First cycle: samples taken after one week (t7) and at the end of the two-week cycle (t15). Second cycle: samples taken after one week (t7.2) and at the end of the two-week cycle (t15.2).

Organic matter removal was evaluated through COD analysis. In the CWs with *P. australis* (Figure 11), at the end of the first week (t7) there was a removal of 79% and 76% for A and L systems, respectively. After two weeks, at the end of the first cycle (t15) removals of 82% for both systems were observed. In the second cycle, significant differences were found between the first and second weeks, 74% and 70% at t7.2 and 89% and 88% at t15.2 for A and L, respectively. For both cycles, no significant differences between substrates were observed.

**Figure 12:**
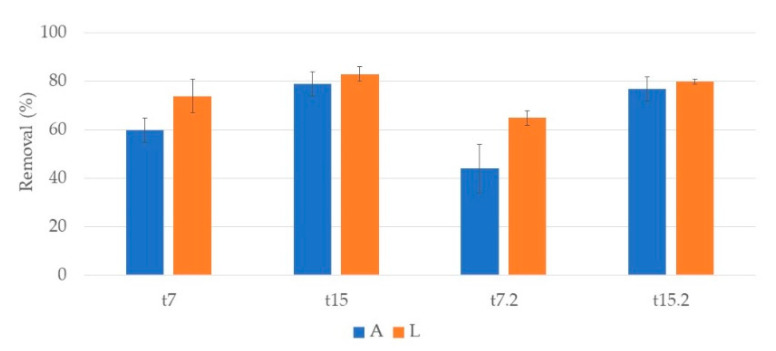
Removal percentage and respective standard deviation (n = 3) of COD, in CWs with *T. latifolia* and expanded clay (A) or lava rock (L) over the two cycles of treatment. First cycle: samples taken after one week (t7) and at the end of the two-week cycle (t15). Second cycle: samples taken after one week (t7.2) and at the end of the two-week cycle (t15.2).

In CWs with *T. latifolia* (Figure 12), at the end of the first week (t7) a removal of 60% and 74% for A and L systems, respectively, was observed. After one week, at the end of the first cycle (t15), a slight removal increase was observed for both systems, 79% and 83% for A and L, respectively. The second cycle showed a similar behavior but with lower removal values, 44% and 65% at t7.2 and 77%, and 80% at t15.2 for A and L, respectively. In general, no significant differences were found between substrates.

## 4. Discussion

The use of reclaimed water is a sustainable alternative to reduce freshwater consumption, being an important part of the concept of the circular economy [39]. Livestock wastewaters are highly contaminated with metals, pharmaceuticals, and other contaminants that need to be removed during treatment if one aims to use this reclaimed water as fertilizer. CWs can be a suitable solution for the removal of toxic contaminants. But the challenge is to maintain the nutritional value of the reclaimed water. To our knowledge, there are no applications of CWs with this purpose. In the present study, high removal rates (69–98%) of metals during livestock wastewater treatment by CWs were observed, being similar to those reported previously [27]. Moreover, metals concentrations after CWs treatment were lower than the maximum values reported in the Portuguese and international legislation for irrigation water [13,14,15]. Lower values than recommended values were achieved for all metals after the treatment, except for Mn, with values slightly higher.

Results indicated that different metals interact differently with the components of CWs, at different paces, resulting in different removal rates over time.

Removal of metals in CWs can occur through different processes, such as uptake by plants, bacteria, and algae, precipitation as insoluble salts, and adsorption to substrates [27]. Studies reported that substrate is considered the primary sink for metals and only a small fraction of the influent loading is removed by accumulation in the aboveground biomass of plants [27,40]. Metals have a positive charge, so are readily adsorbed, complexed, and bound with suspended particles, settling in the substrate [41]. Combinations of different layers of substrates and porous materials can have a positive impact on the removal of contaminants. Commonly used substrates in CWs are, for instance, clay, gravel, volcanic (lava) gravel, and sand [42]. In this study, sand was used as support media for the plant roots. Underneath, a layer of a more porous substrate was included, testing, in this case, two materials, expanded clay and lava rock. Mn was the only metal showing significant accumulation in sand by the end of the experiment in CWs of both plants. In fact, it has been reported that fine grains, with silt or clay, tend to favor metal adsorption [43]. According to ISO 14688-1:2002, the sand used in the CWs setup is considered a coarse sand (~0.7 mm), with no fine grains, which may explain the non-retention of metals in this substrate.

As mentioned, lava rock and expanded clay were used as substrates, both having high absorption capacity, porosity, and good ion exchange capacity. These substrates have been reported to be efficient in removing metals from contaminated water in CWs, so metals might have been adsorbed to these substrates. However, metals were not determined in lava rock and expanded clay as their recovery at the end of the experiment (by dismantling the systems) was not possible. Still, no significant differences were found in metal removals between substrates in CWs with *T. latifolia*. In CWs with *P. australis*, significant differences were only found for Fe and Mn, being A systems more efficient for Mn and L systems more efficient for Fe, showing the different interactions that different metals can have within CWs. But metals removals were high for either substrate. So, both substrates are suitable for metal removal from livestock wastewater.

Removal of metals from wastewaters in CWs may also occur by plant uptake. Several studies reported the efficiency of *P. australis* and *T. latifolia* to accumulate metals [10,27,44,45], being two of the most used plants in CWs [30]. Different factors can influence metal uptake by plants, such as clay content, cation exchange capacity, soil pH, organic matter content, and the presence of other ions [45]. Additionally, the potential of metal uptake varies largely between plant species, being important to do a proper selection of the plant species for specific target metals [45]. Therefore, in the present study removal efficiency of *P. australis* and *T. latifolia* were compared. Accumulated metal can be distributed through different plant tissues. Thus, metals were determined in the different tissues of the two plant species used: root, rhizome, stem, and leaves for *P. australis* and root, rhizome, and leaves for *T. latifolia* (this plant does not have a stem structure). In the present study, *P. australis* showed accumulation of Mn in all plant tissues, Zn in stems and leaves, and Fe only in leaves. For all other metals, levels by the end of the experiment were identical or lower to those initially detected. *T. latifolia* showed high initial concentrations of all metals, with levels decreasing by the end of the experiments, resulting in no accumulation of metals in any plant tissues. In fact, plants appear to have released some metals. Initial concentrations in *P. australis* were similar to previous studies, but initial concentrations of *T. latifolia* were higher than values reported previously [10,26,46]. So, considering the high concentration of metals already present in plants and also, considering the high biomass of each macrophyte in each bed system, the metal concentration removed from the wastewater represented a low value that did not result in significant accumulations in plant tissues. Nevertheless, systems with either plant removed high amounts of all metals, the only exception being Ni. So, either plant is suitable to be included in CWs for the removal of metals from livestock wastewater.

CWs systems were able to remove a high amount of metals from livestock wastewater without saturation of the system as no significant amounts of metals were accumulated in plant tissues or sand substrate. In addition, no major differences were observed between cycles of treatment, despite the usual cumulative effect in these systems, which also suggests that the systems were not saturated. CWs retention time usually varies between 4 and 15 days but studies showing a shorter retention time can also be effective [47], including in the removal of metals. For instance, removals of 63–93% of different metals have been reported in CWs in a retention time of 3 days [40]. In the present case, metal removals did not show a major difference between 7- and 15-days treatments. Moreover, high removal occurred already on the first days of CW treatment, increasing in most cases only slightly over time, indicating that metals were mostly retained in CW system components as soon as the wastewater contacted them. So, a shorter retention time might be enough for the removal of metals, which allows greater retention of nutrients and organic matter.

One of the main constituents of livestock wastewater is organic matter. Results obtained of COD showed high removal rates (44–89%) of organic matter from the wastewater during CWs treatment. Previous studies have shown the potential of CWs for organic matter removal with identical or slightly higher removal rates, including from livestock wastewater [8,23,24,25]. Removal of organic matter involves biological, chemical, and physical processes, such as separation and retention in substrate allowing a proliferation of microorganisms and biodegradation [8]. Plants also lead to higher removal of organic matter by increasing microbial activity [23]. So, different substrates and/or different plants can promote different microbial communities which can influence organic matter removal. However, in the present study, no differences were found between CWs with expanded clay and CWs with lava rock. Results also suggest that both CWs with *P. australis* and CWs with *T. latifolia* are efficient in removing organic matter, with no significant differences between them.

Other main constituents of livestock wastewater are the nutrients nitrogen and phosphorus. Nitrogen can be present in different forms, namely in the form of ammonium, nitrite, and nitrate ions. The major mechanisms of nitrogen removal from wastewater in CWs are microbial processes such as nitrification and denitrification. Nitrification is the biological oxidation of ammonium to nitrite and then nitrate in a sequential reaction. Denitrification is the process in which nitrate is transformed in dinitrogen via intermediates nitrite, nitric oxide, and nitrous oxide [8]. For phosphorus, major removal processes involve fixation by iron and aluminum in the CW’s substrates [48]. Along with these, there are other processes involved in nutrient removal, such as ammonium volatilization, plant and microbial uptake, sorption, desorption, burial, and leaching among others [8]. So, both substrate type and plant species can influence nutrients removal.

In the present study, ammonium ion removal was slightly lower than in previous studies in similar CWs [8,21]. In CWs with *P. australis*, ammonium ion removals reached ca. 85%, showing high removals in the first days, without significant differences between substrates. In CWs with *T. latifolia*, ammonium ion removals were also similar during the first 7 days, increasing to ca. 94% after 15 days of treatment. Both substrates and plants can stimulate different microbial communities that could influence the removals of ammonium, but in the present study, no significant differences were found between systems with different plants or substrates. Removals of nitrites and nitrates ions showed high variability in their removals. This variation can be related to the balance among the different processes of nitrification, denitrification, and assimilation by plants. Daily recirculation of wastewater also enhances nitrification processes, and consequently removals of ammonium [49], but in the present study, recirculation might not be always efficient. Additionally, in some of the microcosms, some anaerobic zones might have occurred leading to variations in removal rates.

The main processes for phosphorus removal are precipitation, sorption, and plant uptake, namely through plant roots. Reported removal rates in similar CWs [8,21] were identical to the ones obtained in this study. In both experiments, removals of phosphate ion showed a tendency to increase over time. In general, no significant differences were found between substrates. But CWs with *P. australis* showed higher removal rates than CWs with *T. lafitolia* (reaching 92% vs. 65%). Although the plant species could have influenced this result, substrate saturation should also be considered as *T. latifolia* were transplanted into CWs with expanded clay and lava rock from CWs with *P. australis*.

High removals of metals were already observed in the first days of CWs treatment, which suggests that a short retention time can be a good option. A shorter retention time can lead to lower removals of nutrients, promoting the use of reclaimed water as fertilizer, although in the present study nutrients removals were already high after only two or three days of CW treatment.

As mentioned, the choice of plants is an important issue in CWs once they mediate important processes. For example, plant metabolic activity releases oxygen into the rhizosphere, promoting the activity of bacteria involved in carbon and nitrogen cycles. The most widely used plants in CWs are cattails (*Typha* spp.) and reeds (such as *Phragmites australis*) [50], being these the plants selected for this study.

Different studies have already reported the suitability of the two plants for metal removal from contaminated wastewater. For instance, Hejna et al. [10] reported that *T. latifolia* accumulates and translocates Zn and Cu from contaminated wastewater into plant tissues, the increase of both metals in plants is related to a decrease of metals in water. Almeida et al. [27] observed also a high removal of different metals (Zn, Cu, Fe, and Mn) from swine wastewater during treatment in CW planted with *P. australis*.

Although the efficiency for metal removal varied with the plant species [51] as well as with the metal itself, removal rates as high as 90% have been reported (e.g., [52]. Similar removal rates were observed in the present study, with removal rates reaching in general >80% for Cd, Cr, Cu, Fe, Mn, Pb, and Zn after 7 or 15 days CW treatment planted with either plant. Previous studies have shown that both *P. australis* and *T. latifolia* are suitable species for phytoremediation, and CWs with these plants can be used for wastewater treatment and be an option for improving irrigation water quality [8,26,33].

Kumari et al. [44] reported that *P. australis* showed higher accumulative capacities for Cu, Cd, Cr, Ni, and Fe than *T. latifolia* but, in the present study, no significant differences in metal accumulation were observed and metal removal rates were identical between the two plants. However, regarding only two days of treatment, these removal rates are only attained in CWs planted with *T. latifolia* (with the exception of Pb with removal rates between 60 and 70%). In CWs planted with *P. australis*, after two treatment days, metal removals rates were ca. 60% (except for Pb with removal ca. 40%). So, CWs planted with *T. latifolia* are more suitable for the aim of this work, that nutrients final concentrations in reclaimed water are maintained for fertilization, after the removal of the toxic contaminants.

The efficiency of metal removal can also vary with the substrate composition. As mentioned, substrate is considered the primary sink for metals accumulation. The selection of a substrate with a high sorption capacity can be an important step in the optimization of CWs performance [53]. Most CWs employ crushed stones, sand, and gravel as substrates not only to support the plant growth but also to act as a filter; however, other substrates such as clay soil, zeolites, shells, and industrial wastes (furnace slag, steel slag, sludge from waste treatment plants) had also been found as efficient filter materials (Arivoli et al., 2015). In the present work, two commonly used substrates were selected, expanded clay and lava rock, to complement the sand used to give support to plant roots.

Calheiros et al. [35] reported that expanded clay aggregates were adequate for the establishment of *T. latifolia*, allowing a high plant propagation level. Moreover, Dordio et al. [53] observed high removals of TSS, COD, NH_4_-N from swine wastewater when using CW with expanded clay substrate and *P. australis* plants and Almeida et al. [8] also observed a similar behavior when using CW with lava rock substrate and *P. australis* plants. In the later system, the CW system also showed high metal removal [27], similar to what was observed in the present study, removal >80%.

Looking at only two days of treatment, metal removal rates were, in general, identical between CWs with expanded clay or lava rock, although slightly higher removals were observed for Fe with lava rock whereas for Mn removals were slightly higher for expanded clay. But for nutrients, namely nitrate and phosphate ions a slightly lower removal was observed when CW had expanded clay. So, both porous substrates can be an adequate option for agricultural wastewater treatment, but expanded clay could be more suitable if one wants lower nutrient removal during a short CW treatment.

## 5. Conclusions

Present results demonstrate both microcosms simulating CWs planted with *P. australis* and planted with *T. latifolia* were able to remove significant amounts of toxic contaminants, such as metals, from livestock wastewater, showing high removal rates after 7 or 15 days of CW treatment. In fact, no difference between plant performance was observed indicating that *P. australis* and *T. latifolia* are appropriate species to be included in CWs for wastewater treatment. However, for a short CW treatment, *T. latifolia* could be a more suitable option as metal rates are already very high after two treatment days.

Results also show that either substrate selected, lava rock and expanded clay, combined with the sand used for plant roots support, contributed to pollutants removal without significant difference between them. So, both substrates can be suitable options for CWs assemblage, although in a short CW treatment expanded clay could be a more suitable choice as it showed slightly lower nutrients removals (nitrate and phosphate ions) in the first days of treatment.

Results also indicate that concentrations of nitrogen and phosphorus decreased during CWs treatment, while still maintaining levels that could be useful for fertilization.

In general, metal concentrations in wastewater treated by the CWs microcosms were below values established in Portuguese and international legislation for irrigation.

Thus, CWs seem to be a valuable and low-cost alternative to treat livestock wastewater so that it can be reused in agriculture, promoting a circular economy.

## Supplementary Materials

The following are available online at https://www.mdpi.com/1660-4601/17/22/8592/s1, Figure S1: (a) Schematic representation of CW microcosm; (b) Picture of real CWs microcosms with *T. latifolia*, Table S1: Initial concentrations (mean and standard deviation, *n* = 3) for each metal in livestock wastewater collected, for the first (t0) and second (t0.2) cycles, Table S2: Initial concentrations (mean and standard deviation, *n* = 3) for each metal in sand and plant tissues, Table S3. Final concentrations at t15.2 and respective standard deviation (*n* = 3) for each metal in sand and plant tissues for the CWs with expanded clay, Table S4. Final concentrations at t15.2 and respective standard deviation (*n* = 3) for each metal in sand and plant tissues for the CWs with lava rock, Table S5: Initial concentrations (mean and standard deviation, *n* = 3) for each nutrient ion in livestock wastewater collected, for the first (t0) and second (t0.2) cycles, Table S6: Legislated values of metals in irrigation wastewater and wastewater discharge in Portuguese and international legislation.
