# Supplementary material for: Livestock Wastewater Treatment in Constructed Wetlands for Agriculture Reuse
Source: Int J Environ Res Public Health. 2020 Nov 19;17(22):8592. doi: 10.3390/ijerph17228592 (PMC7699426; doi:10.3390/ijerph17228592)
Supplement: Supplementary file 1 [file ijerph-17-08592-s001.pdf]

International Journal of
Environmental Research and Public Health

Supplementary Material—Dias et al.

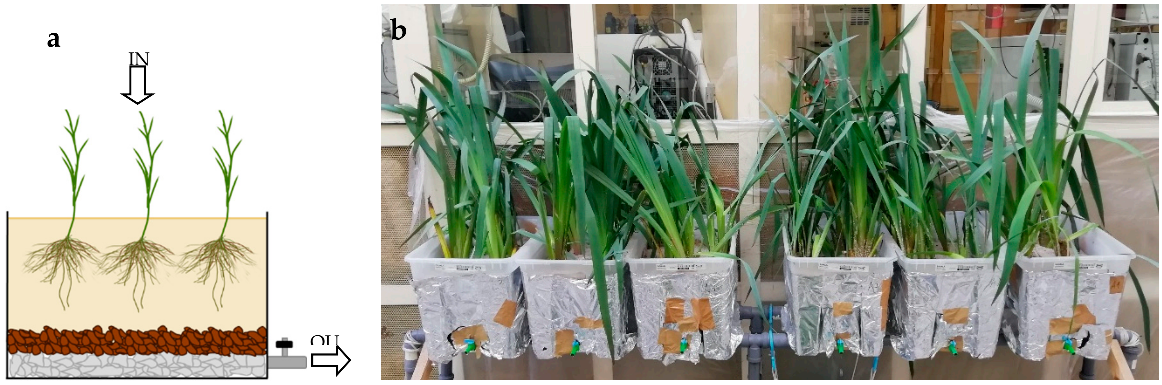

Figure S1. (**a**) Schematic representation of CW microcosm; (**b**) Picture of real CWs microcosms with *T. latifolia*. The wastewater is changed every 15 days.

Table S1. Initial concentrations and respective standard deviation (*n* = 3) for each metal in livestock wastewater collected, for the first (t0) and second (t0.2) cycles.

	P. australis		***T. latifolia***	
	**t0**	**t0.2**	**t0**	**t0.2**
Fe (mg/L)	9 ± 1	13.0 ± 0.1	6 ± 2	2.6 ± 0.1
Zn (mg/L)	4.2 ± 0.1	4.5 ± 0.1	4 ± 1	2.2 ± 0.3
Mn (mg/L)	3.3 ± 0.1	3.8 ± 0.1	2 ± 1	1.1 ± 0.1
Cu (mg/L)	2.0 ± 0.1	2.50 ± 0.03	1.3 ± 0.5	0.7 ± 0.1
Ni (µg/L)	126 ± 23	124 ± 12	83 ± 22	59 ± 5
Cr (µg/L)	75 ± 6	85 ± 2	77 ± 16	56 ± 2
Pb (µg/L)	90 ± 14	63 ± 38	49 ± 21	50 ± 34
Cd (µg/L)	12 ± 3	19 ± 1	13 ± 7	5 ± 1

Table S2. Initial concentrations and respective standard deviation (*n* = 3) for each metal in sand and plant tissues.

		P. australis				***T. latifolia***		
	**Sand**	**Roots**	**Rhizomes**	**Stems**	**Leaves**	**Roots**	**Rhizomes**	**Leaves**
Fe (mg/g)	0.44 ± 0.01	3.1 ± 0.5	1.4 ± 0.5	0.4 ± 0.2	0.4 ± 0.1	3.9 ± 0.1	0.9 ± 0.1	1.9 ± 0.4
Zn (µg/g)	< 4	67 ± 5	26 ± 2	10 ± 3	10 ± 1	303 ± 20	198 ± 14	86 ± 20
Mn (µg/g)	< 4	21 ± 3	8 ± 2	10 ± 1	11 ± 1	147 ± 6	46 ± 3	83 ± 15
Cu (µg/g)	< 4	15.2 ± 0.4	7 ± 1	2.4 ± 0.1	2.7 ± 0.5	80 ± 8	45 ± 4	21 ± 2
Ni (µg/g)	< 0.2	4.8 ± 0.5	3 ± 1	< 1	< 1	7 ± 1	1.7 ± 0.1	1.9 ± 0.2
Cr (µg/g)	0.9 ± 0.3	3.1 ± 0.5	4 ± 1	2.0 ± 0.3		14 ± 3	3 ± 1	7 ± 1
Pb (µg/g)	13 ± 8	16 ± 2	6 ± 2	< 5	< 5	9 ± 3	1.9 ± 0.5	5.2 ± 0.5
Cd (µg/g)	0.7 ± 0.1	0.8 ± 0.5	1.1 ± 0.9	1.4 ± 0.3	1.2 ± 0.2	0.38 ± 0.05	0.16 ± 0.02	0.21 ± 0.03

Table S3. Final concentrations at t15.2 and respective standard deviation ($n = 3$) for each metal in sand and plant tissues for the CWs with expanded clay.

		P. australis				*T. latifolia*		
	**Sand**	**Roots**	**Rhizomes**	**Stems**	**Leaves**	**Roots**	**Rhizomes**	**Leaves**
Fe (mg/g)	0.4 ± 0.1	4 ± 1	0.5 ± 0.2	0.10 ± 0.04	1.6 ± 0.2	3 ± 1	0.5 ± 0.3	0.15 ± 0.05
Zn (µg/g)	4 ± 1	79 ± 12	21 ± 2	41 ± 4	25 ± 4	298 ± 77	104 ± 24	67 ± 6
Mn (µg/g)	4 ± 1	58 ± 8	18 ± 3	19 ± 3	27 ± 3	129 ± 58	24 ± 9	27 ± 3
Cu (µg/g)	< 4	14 ± 1	2.0 ± 0.4	1.8 ± 0.4	6 ± 1	54 ± 14	38 ± 5	7 ± 3
Ni (µg/g)	2 ± 1	3.7 ± 0.5	0.9 ± 0.2	0.4 ± 0.2	0.9 ± 0.2	8 ± 2	1.7 ± 0.4	2 ± 1
Cr (µg/g)	1.4 ± 0.3	1.9 ± 0.4	0.5 ± 0.2	0.9 ± 0.4	5 ± 2	10 ± 3	0.8 ± 0.4	0.7 ± 0.3
Pb (µg/g)	1.0 ± 0.3	4 ± 1	0.9 ± 0.5	0.7 ± 0.5	1.8 ± 0.4	13 ± 4	1.0 ± 0.3	0.5 ± 0.2
Cd (µg/g)	< 0.05	0.08 ± 0.02	0.06 ± 0.03	0.04 ± 0.02	0.03 ± 0.01	2 ± 1	0.12 ± 0.02	0.19 ± 0.05

Table S4. Final concentrations at t15.2 and respective standard deviation ($n = 3$) for each metal in sand and plant tissues for the CWs with lava rock.

		P. australis				*T. latifolia*		
	**Sand**	**Roots**	**Rhizomes**	**Stems**	**Leaves**	**Roots**	**Rhizomes**	**Leaves**
Fe (mg/g)	0.5 ± 0.1	3 ± 1	0.8 ± 0.4	0.12 ± 0.07	1.0 ± 0.4	2 ± 1	0.4 ± 0.3	0.2 ± 0.1
Zn (µg/g)	< 4	61 ± 9	21 ± 5	40 ± 7	31 ± 4	191 ± 65	125 ± 34	69 ± 15
Mn (µg/g)	7 ± 1	58 ± 10	23 ± 6	20 ± 6	28 ± 5	109 ± 52	33 ± 10	39 ± 12
Cu (µg/g)	< 4	9 ± 2	2 ± 1	< 1.4	3 ± 1	43 ± 11	41 ± 18	9 ± 3
Ni (µg/g)		4 ± 1	1.2 ± 0.4	0.3 ± 0.1	1.4 ± 0.6	8 ± 5	1.7 ± 0.6	2.1 ± 0.4
Cr (µg/g)	0.9 ± 0.6	3 ± 1	0.9 ± 0.4	0.9 ± 0.4	3 ± 1	8 ± 4	0.8 ± 0.5	1.0 ± 0.7
Pb (µg/g)	1.7 ± 0.5	4 ± 1	1.1 ± 0.4	0.3 ± 0.2	1.8 ± 0.3	10 ± 2	0.9 ± 0.5	0.4 ± 0.2
Cd (µg/g)	< 0.05	0.12 ± 0.08	0.06 ± 0.04	0.03 ± 0.02	0.06 ± 0.02	0.7 ± 0.2	0.2 ± 0.1	0.15 ± 0.04

Table S5. Initial concentrations and respective standard deviation ($n = 3$) for each nutrient ion in livestock wastewater collected, for the first (t0) and second (t0.2) cycles.

	P. australis		*T. latifolia*	
	**t0**	**t0.2**	**t0**	**t0.2**
NH_4 (mg/L)	675 ± 11	872 ± 56	721 ± 5	564 ± 43
NO_2 (mg/L)	2.6 ± 0.1	1.08 ± 0.03	5.3 ± 0.4	1.3 ± 0.2
NO_3 (mg/L)	15 ± 1	29 ± 5	78 ± 16	8 ± 1
PO_4^- (mg/L)	171 ± 2	220 ± 6	122 ± 2	110 ± 34

Table S6. Legislated values of metals in irrigation waste and wastewater discharge in Portuguese and international legislation.

	Portuguese Legislation			US EPA
	Irrigation Water		Wastewater Discharge	Irrigation Water
	**Reccomended**	**Maximum**		
Fe (mg/L)	5.0		2.0	5.0
Zn (mg/L)	2.0	10.0		2.0
Mn (mg/L)	0.2	10	2.0	0.2
Cu (mg/L)	0.2	5.0	1.0	0.2
Ni (mg/L)	0.5	2.0	2.0	0.2
Cr (mg/L)	0.1	20	2.0	0.1
Pb (mg/L)	5.0	20	1.0	5.0
Cd (mg/L)	0.01	0.05	0.2	0.01

© 2020 by the authors. Submitted for possible open access publication under the terms and conditions of the Creative Commons Attribution (CC BY) license (http://creativecommons.org/licenses/by/4.0/).
